# Practical and reliable FRET/FLIM pair of fluorescent proteins

**DOI:** 10.1186/1472-6750-9-24

**Published:** 2009-03-25

**Authors:** Dmitry Shcherbo, Ekaterina A Souslova, Joachim Goedhart, Tatyana V Chepurnykh, Anna Gaintzeva, Irina I Shemiakina, Theodorus WJ Gadella, Sergey Lukyanov, Dmitriy M Chudakov

**Affiliations:** 1 Shemyakin and Ovchinnikov Institute of Bioorganic Chemistry RAS, Miklukho-Maklaya 16/10, 117997 Moscow, Russia; 2 Swammerdam Institute for Life Sciences, Section of Molecular Cytology, Centre for Advanced Microscopy, University of Amsterdam, Kruislaan 316, NL-1098 SM, Amsterdam, the Netherlands; 3 Evrogen JSC, Miklukho-Maklaya 16/10, 117997 Moscow, Russia

## Abstract

**Background:**

In spite of a great number of monomeric fluorescent proteins developed in the recent years, the reported fluorescent protein-based FRET pairs are still characterized by a number of disadvantageous features, complicating their use as reporters in cell biology and for high-throughput cell-based screenings.

**Results:**

Here we screened some of the recently developed monomeric protein pairs to find the optimal combination, which would provide high dynamic range FRET changes, along with high pH- and photo-stability, fast maturation and bright fluorescence, and reliable detection in any fluorescent imaging system. Among generated FRET pairs, we have selected TagGFP-TagRFP, combining all the mentioned desirable characteristics. On the basis of this highly efficient FRET pair, we have generated a bright, high contrast, pH- and photo-stable apoptosis reporter, named CaspeR3 (Caspase 3 Reporter).

**Conclusion:**

The combined advantages suggest that the TagGFP-TagRFP is one of the most efficient green/red couples available to date for FRET/FLIM analyses to monitor interaction of proteins of interest in living cells and to generate FRET-based sensors for various applications. CaspeR3 provides reliable detection of apoptosis, and should become a popular tool both for cell biology studies and high throughput screening assays.

## Background

During the last decade genetically-encoded sensors on the basis of FRET (Förster Resonance Energy Transfer) between fluorescent proteins have become popular instruments to study kinetics and localization of different pathways inside living cells [1,2]. However, their application is limited by relatively low dynamic range (donor/acceptor emission ratio change), which is limited, in its turn, by FRET efficiency. In addition, spectral separation can be problematic due to pronounced cross-talks characteristic for the traditional cyan and yellow FRET partners.

Recent development of orange, red and far-red monomeric fluorescent proteins drastically enriched the palette of available genetically encoded FRET pairs [3-8]. Some of the novel combinations available can provide higher FRET efficiency and more reliable spectral separation of the donor and acceptor fluorescence. Shifting the wavelengths of FRET pairs towards the red part of the spectrum reduces input of cellular autofluorescence and generally increases the FRET efficiency due to increased R_0 _values [2,9].

However, the choice of the best appropriate pair is not obvious, both due to the drawbacks found for some of the newly developed orange and red fluorescent proteins and due to unpredictable weak interactions between donor and acceptor, that can lead to enhanced or impaired FRET, depending on the resulting orientation of chromophores. Technical limitations of available microscopy software and hardware further complicate the choice. The lack of comparative information hampers development of FRET-based applications and development of high contrast (i.e., reliably reporting) fluorescent sensors, required for the sensitive studies of molecular biology of cell and for the reliable high throughput and high content screening assays [1].

## Results and discussion

### Properties of the TagGFP-TagRFP pair

In order to identify the preferable FRET pair consisting of recently generated monomeric fluorescent proteins, we screened the palette of Tag proteins (Evrogen JSC). By directly comparing the amplitude of fluorescence before and after separation of fluorescent proteins (see Additional file 1), the TagGFP-TagRFP pair demonstrated the highest dynamic range among tested FRET pairs, and was further characterized in more detail.

**Figure 1:**
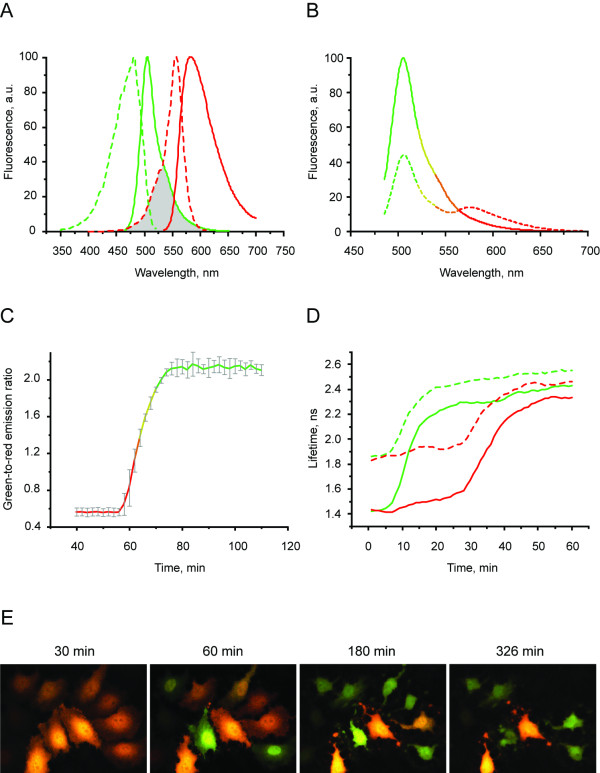
**Spectral response and caspase sensing of CaspeR3**. A. Excitation (dashed lines) and emission (solid lines) spectra for TagGFP (green) and TagRFP (red). Spectral overlap is filled with gray. B. Emission spectra of CaspeR3 before (dashed line) and after digestion by Caspase 3 (solid line). C. Green-to-red emission ratio change of CaspeR3 upon staurosporine-induced apoptosis. Approximately 40–50 min after staurosporine infusion, cells demonstrated pronounced changes fluorescence signal ratio. Emission ratio shown for 5 cells, time point aligned to the median of ratio changes, individual for each cell. Excitation at 488 nm, emission was detected at 500–530 nm and 560–600 nm. D. TagGFP fluorescence lifetime τ_φ _(solid lines) and τ_M _(dashed lines) changes for CaspeR3 during staurosporine-induced apoptosis. Excitation was at 488 nm and donor fluorescence emission was passed through a 500–530 nm bandpass filter. E. Two channel fluorescence imaging of CaspeR3 upon staurosporine-induced apoptosis in HeLa cells. Time, in minutes, is shown after staurosporine infusion.

TagGFP (Evrogen JSC) and TagRFP [7] are bright monomeric fluorescent proteins with excitation/emission peaked at 482/505 nm and 555/584 nm, respectively. The high fluorescence quantum yield of TagGFP along with the high molar extinction coefficient of TagRFP and excellent overlap of donor emission and acceptor excitation spectra result in highly effective FRET (Fig. 1A, B). The Förster radius (calculated using standard methods, see Additional file 1) for FRET between TagGFP and TagRFP is 5.74 nm, being significantly higher than that of the TagGFP-mCherry couple of 5.28 nm. At the same time, since TagGFP and TagRFP emission peaks are spaced by as much as 79 nm, the emission signal for these two proteins can be reliably separated in any imaging system. High pH-stability (pKa 4.7 for TagGFP and 3.8 for TagRFP) makes this pair a reliable pH-independent reporter, and allows to employ it for imaging in acidic organelles.

The excitation wavelength required to visualize FRET changes of the TagGFP/TagRFP pair by ratio-imaging is provided by an ordinary FITC/GFP excitation filter or ubiquitous 488 nm laser line, and the two emission signals are acquired using a 500–530 nm (FITC/GFP emission filter) bandpass filter and a 560–600 nm bandpass filter (Cy3/DsRed emission filter) or a 560LP longpass filter, or the like. As can be inferred from Fig. 1A, the direct acceptor (TagRFP) excitation at 488 nm is marginal, giving rise to an almost pure donor (TagGFP) emission spectrum in the absence of FRET (see Fig. 1B, solid line).

### Caspase 3 sensor

On the basis of this pair of fluorescent proteins we generated a bright, high contrast, pH- and photo-stable FRET-based apoptosis reporter, named CaspeR3 (**Casp**ase **3 R**eporter), constructed of TagRFP and TagGFP connected via 17 a.a. peptide linker containing caspase-3-cleavable motif DEVD [10] (see Additional file 1 for the construct scheme).

The construct was expressed in *E. coli *and demonstrated bright fluorescence after overnight growth at 37°C and purification. Further incubation of a sample did not led to essential changes of fluorescence spectra or brightness, indicating profound maturation of both proteins. The cleavage of a sensor by activated form of the caspase 3 resulted in separation of two fluorescent proteins and elimination of FRET. Direct monitoring of the donor/acceptor emission ratio demonstrated up to 5-fold ratio changes upon cleavage of CaspeR3 by recombinant caspase 3 (BioCat GmbH)*in vitro *(Fig. 1B and Additional file 1). The increase in donor fluorescence intensity was at least 2-fold corresponding to a FRET efficiency of at least 50%. Subsequently, we tested CaspeR3 responsiveness in transiently transfected HeLa cells. Living cells were monitored at 37°C with Leica SP2 confocal microscope (excitation using 488 nm laser line, emission collected at 500–530 nm and 560–650 nm). The fluorescence was evenly distributed in the cytosol and nucleus with no aggregation or non-specific localization observed. Importantly, both green and red signals were reliably stable upon blue excitation in various irradiation conditions for hours. No reversible or irreversible fluorescence bleaching or photoconversion were observed.

Apoptosis was induced by treatment with 2 μM staurosporine (Calbiochem) Approximately 40–50 min after staurosporine infusion (incubation at 37°C), cells demonstrated rapid (within 10 min) and pronounced changes in green-to-red fluorescence signal ratio, indicating activation of caspase 3. Later these cells demonstrated characteristic membrane blebbing. The average contrast in living cells (calculated as donor/acceptor emission ratio change for 5 cells, time point aligned to the median of ratio changes, individual for each cell) reached 3.8-fold (Fig. 1C).

### FLIM

One of the most powerful and quantitative approaches to measure FRET changes is fluorescence lifetime imaging (FLIM), which measures the effect of the acceptor on the excited state lifetime of the donor. If the acceptor is in close proximity, the lifetime is reduced. The reduction of the fluorescence lifetime is a kinetic parameter that can be determined independently of probe concentration, microscope optical path and moderate levels of photobleaching. Therefore, the reduction of the donor lifetime is an extremely robust and quantitative estimate of FRET efficiency that is directly proportional to the amount of uncleaved substrate. We performed FLIM measurements of the non-fused TagGFP and the TagGFP within the CaspeR3 sensor in living cells. These experiments demonstrated substantial differences in the detected fluorescence lifetimes (Table 1). Accordingly, upon staurosporine-induced apoptosis, fluorescence lifetime of TagGFP within CaspeR3 changed dramatically, switching from 1.5 ns to 2.5 ns (Fig. 1D and Additional file 2).

**Table 1:** FLIM measurements for the TagGFP and TagGFP-TagRFP pair

Construct	n^1^	τ_φ _[ns]^2^	τ_M _[ns]^3^	Eτ_φ _[%]^4^	Eτ_M _[%]^4^
TagGFP	20	2.55 ± 0.05	2.64 ± 0.04	-	-
TagGFP-TagRFP	23	1.57 ± 0.07	1.98 ± 0.07	38	24

^1^n number of cells from which the lifetime was calculated

^2^τ_φ _average phase lifetime ± standard deviation

^3^τ_M _average modulation lifetime ± standard deviation

^4^E average FRET efficiency calculated from τ_φ _or τ_M_

The FRET efficiency of the uncleaved CaspeR3 (38% based on the phase lifetime) is among the highest measured by FLIM and compares favorably to a red-to-green caspase sensor reported previously, with a FRET efficiency of 25% [11]. Since the FRET efficiency of the cleaved substrate is zero, the dynamic range of the sensor is rather high, indicating that TagGFP-TagRFP FRET pair will be an excellent tool for the high content FLIM based screenings on living cells.

## Conclusion

Most reported FRET indicators are based on historically first (BFP)CFP/YFP pairs [12-15]. However, these FRET pairs are not really the most convenient and effective. Indeed, spectral separation of overlapping cyan and yellow emission spectra can never be complete, and use of narrow band-pass filters results in dramatic loss of emission. Besides, the relative high levels of autofluorescence in blue-cyan region of visible spectrum and phototoxicity using near-UV excitation further complicates their application.

Reported FRET pairs containing red fluorescent acceptors suffer from tetramerization and demonstrate lower contrast [16,17]. The closest competitor of CaspeR3 is a high contrasting caspase-3 indicator MiCy-mKO [3], However, it was observed that mKO converts from orange to green fluorescent form upon blue light illumination [9], that can hamper interpretation of FRET changes in this and other mKO-based techniques.

The high extinction coefficient of TagRFP makes it a preferable FRET acceptor for the green fluorescent proteins. It is of note that the actual FRET efficiency is an inverse 6^th ^power distance dependency, leading to a quick drop of the detected FRET efficiency at donor-acceptor proximities above R_0_. For instance, under similar conditions, we expect that, given the Förster radii 5.28 nm and 5.74 nm for TagGFP-mCherry and TagGFP-TagRFP respectively, the latter would display 1.4-fold more FRET than a sensor comprised of the TagGFP-mCherry pair.

The superior Förster radius is caused by an 1.5-fold increased spectral overlap for the TagGFP-TagRFP pair as compared to the TagGFP-mCherry pair. While not contributing to the FRET efficiency, the significantly increased quantum yield of TagRFP is highly beneficial for acceptor-based ratiometric FRET studies.

Altogether, the combined advantages of the TagGFP-TagRFP make it the FRET pair of choice both for the ratiometric FRET analyses and FLIM assays to monitor interaction of proteins of interest in living cells, as well as to generate high contrast FRET-based genetically encoded sensors for various analites and protein activities.

## Methods

### Cloning and gene construction

For bacterial expression, corresponding PCR-amplified fluorescent proteins genes were sequentially cloned in frame into the pQE30 vector (Qiagen) using *BamHI/KpnI *and *KpnI/HindIII *restriction sites. For expression in eukaryotic cells, the CaspeR3 construct was swapped for TurboGFP within the pTurboGFP-N vector (Evrogen) using pre-introduced *AgeI/NotI *restriction sites.

### Protein expression and in vitro spectroscopy

Proteins fused to the N-terminal polyhistidine tag were expressed in *E. coli *XL1 Blue strain (Invitrogen). The bacterial cultures were centrifuged and the cell pellets re-suspended in 20 mM Tris-HCl, 100 mM NaCl, pH 7.4 buffer and lysed by sonication. The recombinant proteins were purified using TALON metal-affinity resin (Clontech) followed by a desalting step over gel-filtration columns (Bio-Rad). A Varian Cary Eclipse Fluorescence Spectrophotometer was used for measuring excitation-emission spectra.

### Fluorescence lifetime imaging

Fluorescence lifetime imaging was performed using the wide-field frequency domain approach on a home-build instrument [18] using a RF-modulated image intensifier (Lambert Instruments II18MD) coupled to a CCD camera (Photometrics HQ) as detector. A 40× objective (Plan NeoFluar NA 1.3 oil) was used for all measurements. The modulation frequency was set to 75.1 MHz. Twelve phase images with an exposure time of 100–200 ms were acquired in a random recording order to minimize artifacts due to photobleaching [19]. An argon-ion laser was used for excitation at 488 nm, passed onto the sample by a 495 nm dichroic mirror and emission light was filtered by a 515/30 nm emission filter. Every FLIM measurement was followed by a reference measurement. The reference was calibrated by averaging three to five FLIM measurements of a 1 mg/ml solution of erythrosine B (cat # 198269, Sigma-Aldrich, Zwijndrecht, The Netherlands) in H_2_O, which has a known short fluorescence lifetime of 0.08 ns [19,20]. From the phase sequence an intensity (DC) image and the phase and modulation lifetime image was calculated using Matlab macros. From this data, the lifetime of individual cells was determined using ImageJ . Subsequently, average phase and modulation lifetimes ( ± standard deviation) were calculated. For the presentation of lifetime maps, a 3 × 3 smooth filter was applied to the lifetime data. The false-color lifetime maps and 1D and 2D histograms were generated by an ImageJ macro.

## Competing interests

TagGFP, TagRFP and CaspeR3 are the property of Evrogen JSC, Moscow, Russia.

SL and DMC have interest in Evrogen JSC.

Vector encoding CaspeR3 will be available to the scientific community via Evrogen JSC.

## Authors' contributions

DS and  EAS generated fusion constructs and analyzed FRET pairs *in vitro *and in living cells. TVC performed cell culturing. AG and IIS performed technical assistance. JG and TWJG performed FLIM experiments and participated in the manuscript preparation. SL and DMC wrote the manuscript.

## Supplementary Material

Additional file 1 **Supplementary figures and data.** The data provided represent the comparison of several TagFPs FRET pairs and details concerning Förster radius calculation. Click here for file

Additional file 2 **Change of CaspeR3 green fluorescence lifetime upon staurosporine-induced apoptosis.** This movie demonstrates that upon staurosporine-induced apoptosis fluorescence lifetime of TagGFP within CaspeR3 changed dramatically, switching from 1.5 ns to 2.5 ns. Click here for file
