# Supplementary material for: Practical and reliable FRET/FLIM pair of fluorescent proteins
Source: BMC Biotechnol. 2009 Mar 25;9:24. doi: 10.1186/1472-6750-9-24 (PMC2678114; doi:10.1186/1472-6750-9-24)
Supplement: Additional file 1 — Supplementary figures and data. The data provided represent the comparison of several TagFPs FRET pairs and details concerning Förster radius calculation. [file 1472-6750-9-24-S1.pdf]

# Practical and reliable FRET/FLIM pair of fluorescent proteins

## Supplementary data

Dmitry Shcherbo[1*], Ekaterina A. Souslova[1*], Joachim Goedhart[2], Tatyana V. Chepurnykh[3], Anna Gaintzeva[3], Irina I. Shemiakina[1], Theodorus W J Gadella[2], Sergey Lukyanov[1] and Dmitriy M. Chudakov[1#]

[1] Shemyakin and Ovchinnikov Institute of Bioorganic Chemistry RAS, Miklukho-Maklaya 16/10, 117997 Moscow, Russia.

[2] Swammerdam Institute for Life Sciences, Section of Molecular Cytology, Centre for Advanced Microscopy, University of Amsterdam, Kruislaan 316, NL-1098 SM, Amsterdam, The Netherlands.

[3] Evrogen JSC, Miklukho-Maklaya 16/10, 117997 Moscow, Russia.

[*] These authors contributed equally to this work.

[#] Corresponding author (chudakovdm@mail.ru, tel. +7 495 429-80-20)

# Supplmentary Figure 1.

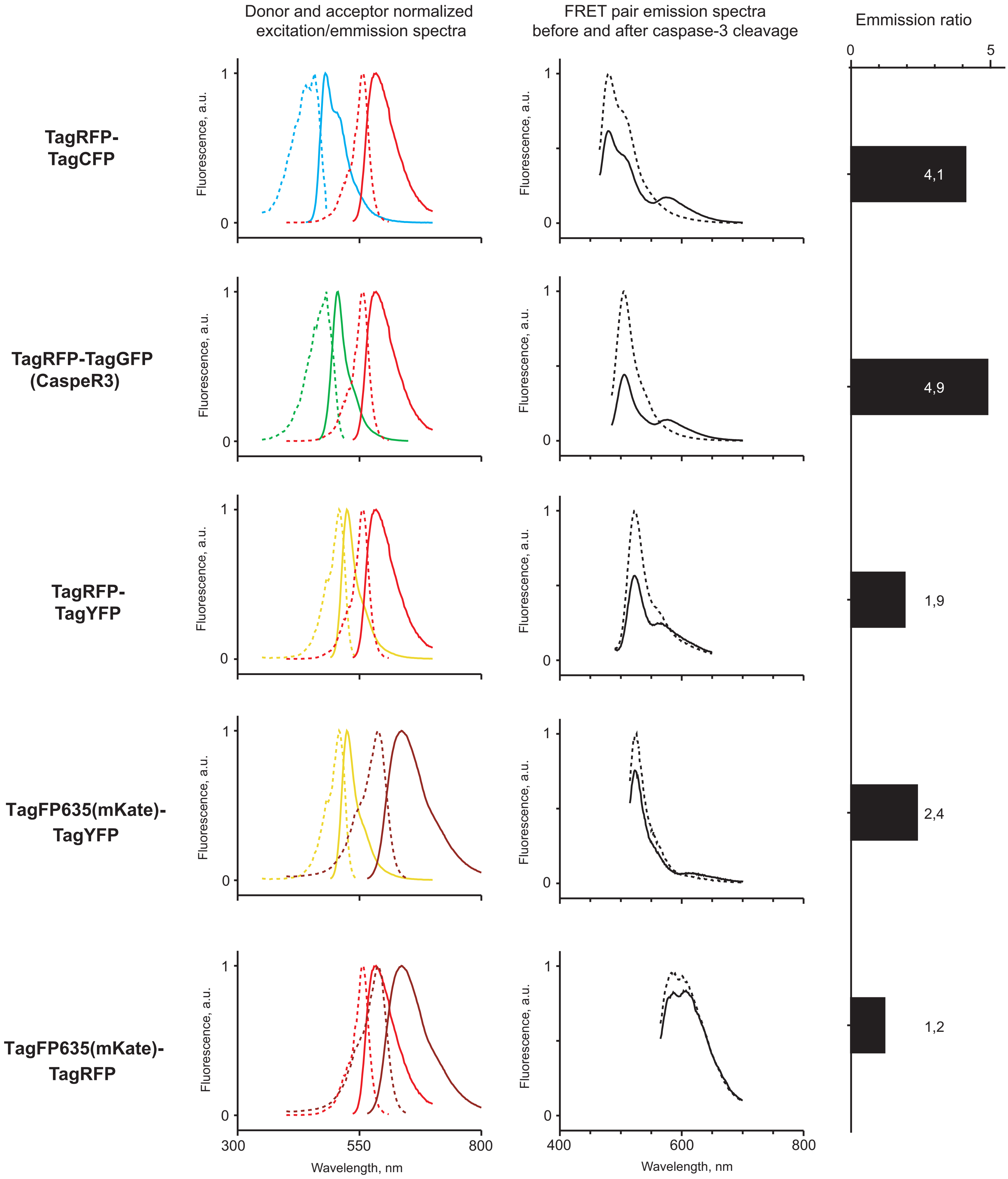

**Supplementary Figure 2.** Protein gel electrophoresis analysis of CaspeR3 before and after cleavage by caspase 3 *in vitro*.

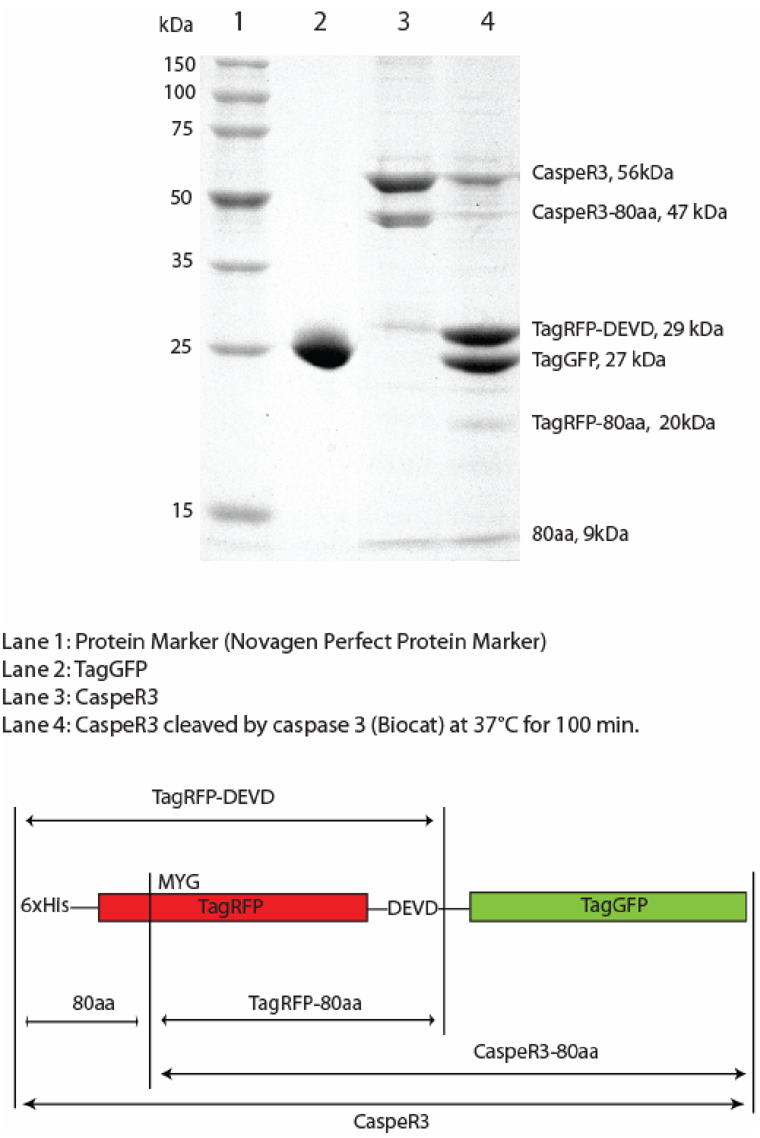


All samples of purified proteins were heated at 95°C for 5 min. Positions of molecular-mass marker are shown on the left (sizes in kDa). Upon heating of samples, red fluorescent proteins that carry DsRed-like chromophore often demonstrate partial fragmentation with a break point just before the chromophore [1, 2]. This well known effect is also observed for CaspeR3 which results in minor additional bands corresponding to 47 kDa (CaspeR3-80aa) and 9 kDa (80aa) fragments for CaspeR3 and 20 kDa (TagRFP-80aa) and 9 kDa (80aa) fragments for the TagRFP cleaved from CaspeR3 by caspase 3. The scheme of the construct and resulting bands are shown on the bottom.

**Supplementary Data 1.** Comparison of TagFPs FRET pairs.

Aiming to find optimal fluorescent partners for FRET we searched among the palette of monomeric fluorescent proteins available from Evrogen JSC, ranging in color from cyan (TagCFP) to far-red (TagFP635) and constructed five chimeric constructs: TagRFP-DEVD-TagCFP, TagRFP-DEVD-TagGFP, TagRFP-DEVD-TagYFP, TagFP635-DEVD-TagYFP and TagFP635-DEVD-TagRFP, cleavable by caspase 3 (**Supplementary Fig 1**). Each of them was tested for maximal dynamic range *in vitro*.

For this, N-terminal-$His_6$-containing recombinant proteins were purified using metal affinity resin Talon (Clontech) and their emission spectra (exited at a donor excitation maximum) were analyzed with Varian Cary Eclipse spectrofluoremeter before and after 1h of incubation at 37°C with (1U/sample) of active human recombinant caspase-3 (BioCat GmbH). Donor/acceptor emission ratio of generated constructs differed from 1.2 for TagFP635-DEVD-TagRFP to 5 for TagRFP-DEVD-TagGFP (**Supplementary Fig. 1**). It was demonstrated that TagGFP was efficient FRET-donor for TagRFP and corresponding TagRFP-DEVD-TagGFP construct was named CaspeR3 (from **Casp**as**e** **3** **R**eporter) and was chosen for further experiments in living cells.

**Supplementary Data 2.** Förster radius calculation.

The Förster radius ($R_0$) for FRET was calculated according to equation 1 and 2 [3, 4]:

$$R_0 = \left(8.79 \times 10^{-5} J q_D n^{-4} \kappa^2\right)^{1/6} \quad \text{(in Å)} \qquad (1)$$

$$J = \int \varepsilon_A(\lambda) f_D(\lambda) \lambda^4 d\lambda \Big/ \int f_D(\lambda) d\lambda \quad \text{(in nm}^4\,\text{M}^{-1}\,\text{cm}^{-1}) \qquad (2)$$

Where $J$ is the spectral overlap integral, $q_D$ is the donor fluorescence quantum yield, $n$ is the refractive index of the medium (assumed 1.3 for the calculation), $\kappa^2$ is the orientation factor (assumed to be $2/3$ corresponding to random donor and acceptor orientation), $\varepsilon_A(\lambda)$ is the (wavelength dependent) extinction coefficient of the acceptor (in $M^{-1}$ $cm^{-1}$), $f_D(\lambda)$ is the (wavelength dependent) donor fluorescence intensity (arbitrary units) and $\lambda$ is the wavelength (in nm).

For the calculation of $R_0$ of TagGFP-TagRFP FRET, $q_D$ was 0.59, and the maximal extinction coefficient of the acceptor $\varepsilon_A(555) = 100{,}000$ M$^{-1}$ cm$^{-1}$ was used to scale the measured TagRFP absorbance spectrum to obtain $\varepsilon_A(\lambda)$. This yielded an overlap integral of $3.24 \times 10^{15}$ nm$^4$ M$^{-1}$ cm$^{-1}$ and an $R_0$ of 57.4 Å. For the calculation of TagRFP -mCherry FRET, the maximal extinction coefficient mCherry $\varepsilon_A(587) = 72{,}000$ M$^{-1}$ cm$^{-1}$ was used to scale the mCherry absorbance spectrum. This yielded an overlap integral of $1.96 \times 10^{15}$ nm$^4$ M$^{-1}$ cm$^{-1}$ and an $R_0$ of 52.8 Å.

Given the Förster equation (3) for FRET [3, 4]:

$$E = \left(1 + (R/R_0)^6\right)^{-1} \qquad (3)$$

in which $E$ is the energy transfer efficiency and $R$ is the donor-acceptor separation, the increased $R_0$ of the TagGFP-TagRFP FRET pair implies that at a donor-acceptor separation of 8 nm this pair displays a 1.5 times higher FRET efficiency than TagGFP-mCherry and a 2.9-fold higher FRET efficiency than ECFP-EYFP (with an $R_0$ of 47.5 Å).

## References:


1. Gross LA, Baird GS, Hoffman RC, Baldridge KK, Tsien RY: **The structure of the chromophore within DsRed, a red fluorescent protein from coral**. *Proc Natl Acad Sci U S A* 2000, **97**(22):11990-11995.
2. Martynov VI, Maksimov BI, Martynova NY, Pakhomov AA, Gurskaya NG, Lukyanov SA: **A purple-blue chromoprotein from Goniopora tenuidens belongs to the DsRed subfamily of GFP-like proteins**. *J Biol Chem* 2003, **278**(47):46288-46292.
3. Stryer L: **Fluorescence energy transfer as a spectroscopic ruler**. *Ann Rev Biochem* 1978, **47**:819-846.
4. Selvin PR: **Fluorescence resonance energy transfer**. *Methods Enzymol* 1995, **246**:300-334.
